# Research on the Training and Management of Industrializing Workers in Prefabricated Building with Machine Vision and Human Behaviour Modelling Based on Industry 4.0 Era

**DOI:** 10.1155/2022/9230412

**Published:** 2022-06-08

**Authors:** Junwu Wang, Yinghui Song, Chunbao Yuan, Feng Guo, Yanru Huangfu, Yipeng Liu

**Affiliations:** ^1^School of Civil Engineering and Architecture, Wuhan University of Technology, Wuhan 430070, China; ^2^Hainan Research Institute, Hainan Research Institute, Sanya 572019, China; ^3^China Construction Seventh Engineering Division Corp Ltd, Shenzhen 518129, China; ^4^School of Art and Design, Zhengzhou University of Light Industry, Zhengzhou 450002, China

## Abstract

As countries around the world pay more and more attention to the sustainable development of the construction industry, the prefabricated building model has become the best construction type to achieve energy conservation and emission reduction. However, the prefabricated building entails higher technical requirements, and the workers involved in the construction must be trained to reduce the risks. For China, where the demographic dividend is gradually disappearing, how to quickly promote the industrializing workers process has become an urgent issue. This research focuses on the training and management of industrializing workers in prefabricated building. First, the facial images of the participants were collected from the actual test data, and the changes of participants' facial expressions were analyzed through multitask convolutional neural network-Lighten Facial Expression Recognition (MTCNN-LFER). The results of the analysis were plugged into the facial expression recognition and evaluation model for industrializing workers training in this research to calculate the weights, and then all the weights were clustered through the improved SWEM-SAM method. The results show the following: (1) the values of objective data were used to judge the participating workers' mastery of each knowledge and to evaluate whether they are qualified. (2) The evaluation results were used to analyze the risk events that may be caused by participating workers.

## 1. Introduction

With the development of global economy and engineering technology, the urbanization and industrialization of each country are also advancing [1], and the advancement of electronic intelligence technology has enabled the world industrial level to move into a New Era of Industry 4.0. In today's world of advocating resource conservation and sustainable development, the traditional construction model consumes a large amount of resources [2, 3] and produces a huge amount of construction waste that causes great damage to the environment. Therefore, the Chinese government has proposed the 14th Five-Year Plan and the long-range goals for 2035 [4], in which prefabricated building model was promoted widely to drive sustainable development of the world environment. Since the prefabricated building model is not yet mature in China, there is a high technical requirement for workers. Besides, as China's population distribution changes, China is gradually entering an ageing society and the demographic dividend is rapidly declining. The Chinese government has proposed to accelerate the process of industrialized workers to ensure sufficient numbers of professionals and high-quality technologies in the construction industry.

According to data released by the Ministry of Housing and Urban-Rural Development of the PRC, this study investigated safety accidents in the construction industry between 2009 and 2020 [5]. 96.17% of accidents with casualties were caused by workers' unsafe behaviours. 284 safety accidents in prefabricated buildings were reported in 2020 alone, with an average casualty rate of 1.63 people per accident [6]. Besides, 72.64% of the economic and legal problems leading to project delays stemmed from the failure of relevant practitioners to fulfil their obligations in accordance with contracts and laws and regulations. Therefore, a strict control of the training and management system for industrialized workers in prefabricated building is of greater significance in origin-tracing of accidents in such projects and can promote the stable and standardized development of the construction industry as a whole [7].

At present, the construction of prefabricated buildings worldwide is through the combination of prefabricated components and cast-in-place technology, the design concept of which is similar to the structural strength of cast-in-place buildings. The main design method is to connect the prefabricated elements to the cast-in-place structure by solid longitudinal reinforcement connections and then technical processes are implemented to the joints of the whole building structure in order to achieve safety and durability that match the architectural design level [8]. As finished products, prefabricated components have high requirements on humidity, storage space, and maintenance methods in terms of production, transportation, and storage. Considering construction, the longitudinal reinforcement connection technology of prefabricated buildings is quite complex with a large number of joints and splicing gaps, and the waterproofing and insulation of walls are technically challenging [9]. Therefore, prefabricated buildings require workers with higher technical skills.

The traditional worker training system is only a two-phase process from training to employment, and the workers are rural migrant workers with a mobile work nature. As these workers are poorly educated generally, they usually rely on “experience” and tend to ignore the correct mechanical operation steps or the standardized and safe construction sequence, which has posed great engineering hidden dangers to the whole project. In recent years, with the advancement of industrialized workers, the relevant training system has incorporated the concept of assessment. But the practice of determining whether the training standards are met only based on the workers' test scores leads to neglect of workers' mastery of knowledge in different situations. It results in redundant training or muddling through the test, which will cause economic losses to the company and even failure to detect safety hazards in time.

In summary, based on the development background of China's construction industry, how to establish a training system with sound risk control for industrialized workers to ensure the quality of the advancement of the industrializing workers and to reduce the incidence of various risks in the construction industry is an urgent problem to be solved at present. This research uses human behaviour modelling as the grassroots theory, adopts a combination of artificial intelligence and machine vision to recognize and analyze the facial expressions of workers undergoing industrializing worker training tests, and decomposes the test results of workers in different emotional contexts, so as to establish a facial expression recognition and evaluation model for industrializing worker training. Then the calculation results were clustered through the improved SWEM-SAM method to evaluate the effects of the worker training and to uncover the hidden risks and hazards in this worker group.

The rest of the research is organized as follows. In Section 2, literature research on basic theory involved in this paper is conducted to find theoretical support.  Section 3 introduces the methods adopted in this research and improvement points. In Section 4, a facial expression recognition and evaluation model for industrializing worker training is established and four different scenarios are analyzed. In Section 5, the actual test data are plugged into the model for analysis.  Section 6 discusses the analysis results, the correctness of the model, and the trends of the interpretation results and concludes the research to provide theoretical directions for further research.

## 2. Literature Review

### 2.1. Human Behaviour Modelling

Human behaviour modelling is an interdisciplinary mathematical modelling method that evaluates what is happening and predicts what will happen through the external behaviour of humans. It has been successfully applied in a number of disciplines. Human behaviour is modelled based on rationality to find optimal solutions [10, 11], but, in practice, much human behaviour is found to deviate from rationality.

Steyn investigated contingency reserves in engineering projects and modelled workers' behaviour at construction sites, so as to use the model to predict contingency reserves and prevent economic overruns of the project and possible shortening of construction period [12]. Meel et al. investigated personnel management in chemical plants and analyzed the management relationship between plant risk calculations and human behaviour patterns to study the correlation between unsafe human behaviour and training the workers received [13, 14]. Zhang et al. analyzed risk factors of man-made accidents in underground engineering by modelling human behaviour from three aspects of safety cognition, behaviour, and physiological states of humans and proposed how to control man-made risk causes [15]. Ronchi modelled dynamic evacuation behaviour of the crowd in fire scenarios by starting with big data analysis. He put forward a theory of management and prevention of disasters caused by crowd gathering by the fitting analysis of large bulk of data of movement patterns of each individual in the crowd and their state performance [16]. Asilian-Mahabadi et al. surveyed a large number of relevant practitioners by studying the prerequisites for unsafe behaviour of front-line workers and managers in civil engineering and discussed the ability of human behaviour models to accurately expose the risk causes of unsafe worker behaviour [17]. van der Kleij and Leukfeldt analyzed the status quo of unsafe behaviour in cyberspace and proposed a new network resilience framework that integrates resilience engineering and human behaviour models. A pilot study of more than 60 small- and medium-sized enterprises in the Netherlands was conducted, the results of which could provide solid recommendations for organizations to better address cyberthreats [18]. Bai and Qian conducted a study on the validity and reliability of the human behaviour cognition factor and found that higher ratings of the people involved in such models had a greater contribution to quality assurance and safety behaviour in construction projects [19].

There are many similar studies that can reflect the existence of a management relationship between human behaviour modelling and accident risk, which means trends of human behaviour can be used to predict the likelihood of risk occurrence. This study aims to identify hidden risks in engineering by analyzing the distribution of workers' test scores.

### 2.2. Emotional Expressions in Facial Expressions

Human facial expressions vary significantly under different emotions and the changes in facial expressions caused by each emotion are basically the same regardless of the country to which the person belongs. Therefore, studies related to emotion communication through facial expressions are mostly associated with human behaviour. Words can sometimes deceive, but a momentary change in facial expressions is difficult to hide.

Suhr et al. started with the rising crime rate of automated bank teller machines. As the machine's sensing system could not accurately determine sabotage, the offenders' facial expressions captured by the camera were identified, especially during the sabotage period, to build a model of facial expressions of emotion, and finally a correlation between facial expressions and insecure behaviour was identified [20]. Pantano collected data on the facial expressions of a large group of consumers when they were engaged in consumer behaviour to build a machine learning model and employed facial expression recognition system to evaluate consumer behaviour and activities, allowing salespeople to guide consumers according to their emotional activities [21]. Israel and Schonbrodt measured the physiological phenomena generated by changes in facial expressions (including facial electromyography, heart rate changes, and brain electrical changes) to create a benchmark test emotion evaluation system based on machine learning algorithms and found that human emotional changes could affect human behaviour [22]. Liu et al. proposed a multilabel and distributed learning approach to analyze students' facial expressions in the classroom and assessed teaching quality by observing respondents' facial expressions. Corresponding improvement suggestions on teaching models were proposed to educational administrators [23]. Monaro et al. used machine learning methods to identify facial microexpressions with the study subject of criminals in interrogation and introduced the concept of speaker cognitive load to determine whether criminals lied to conceal the truth during questioning with the help of machine vision. Given the same control group experiment, judgment by facial expressions was correct 78% of the time, compared to the 57% of human judges [24]. In the healthcare industry, Altameem and Altameem developed a multimodal visualization analysis method by detecting facial expressions on patients to provide emergency treatment for patients through facial expression changes caused by adverse emotions [25]. De la Torre-Luque et al. found that emotion recognition is a key process of social cognition which reflects a person's maximal social adaptability. By comparing the facial expressions of a great number of normal people with those of psychopaths, a theoretical model for emotional decoding of human behaviour and facial expressions was developed [26]. Riquelme et al. collected a large amount of facial expression data of drivers and extracted feature codes to develop a driver fatigue monitoring system, which detects the driver's mouth aspect ratio, blinking frequency, and head tilt angle to determine whether the driver is in fatigue and alerts control staff in time, so as to avoid traffic accidents [27]. In a study on classroom teaching, Schneider et al. found that facial expressions are expressions of mental activity and that different facial expressions have a greater impact on learning efficiency. The analysis of the emotional state of facial expressions provides effective suggestions for educational administrators to improve students' learning efficiency [28].

Through the above scholars' research, it is found that changes in facial expressions can convey the inner activities of human beings, which is a great aid to judging the mental state of the test subjects.

### 2.3. Industrializing Worker Training

“Industrializing workers” refers to, in China where the demographic dividend is gradually disappearing, the process by which construction worker teams, mainly migrant workers, are reeducated, assessed, and qualified to engage in industrial production with long-term work effectiveness. With 200 million mobile migrant workers in China, migrant workers need to receive instruction and education in industrial production ideas, science and technology, the use of wearable devices, and efficient and safe workforce regulations to become industrializing workers.

He's study found that vocational education occupies a greater proportion of worker industrialization, suggesting a connection model that combines higher vocational education and industrializing worker training in conjunction with the talent cultivation models in construction vocational colleges [29]. Wang and Ji conducted a research on the demand for construction workers in different development stages of construction industrialization, analyzed the differences between construction workers in developed countries and in China in various aspects, and pointed out the significance of safety education for industrializing workers and implementation of labor security system on promoting industrializing workers [30]. In a study on the risk cognition of Spanish workers, Rodriguez-Garzon et al. found that the industrial transformation efficiency of workers was related to working population mobility. By aggregation and clustering analysis, it was found that the worker groups who received multiple trainings had stronger risk perception ability [31]. In the industrial transformation of workers in the construction of a laboratory at the Graz University of Technology, Karre et al. found that the key to transformation lied in supervision system and technical training should be strengthened [32]. Ke analyzed the complexity of the evolution from migrant workers to industrializing workers in the construction industry in China on the basis of CAS theory and made recommendations based on an agent-based modelling [33]. Hatami and Kakavand deemed that behaviour modification plays an indispensable part in worker industrialization and proposed the PRECEDE-PROCEED approach to evaluate the educational intervention effectiveness of occupational safety training on industrializing workers. As a result, the programme greatly reduced unsafe behaviour [34]. Radhakrishnan et al. combined the industrializing worker training system with artificial intelligence by using IVR technology to simulate accidents in a virtual environment and transmit the perception data of the trainers [35]. Cao et al. adopted a CHAID decision tree and chi-square analysis from the aspect of construction safety to explore the primary and secondary influencing factors and unrelated factors in education and training for industrializing workers. A five-factor method for training effectiveness was proposed [36].

Most of the previous studies have applied methods of traditional education industry to industrializing worker training, exploring the potential correlation between worker training effects and the reduction of accident probability. Combined with previous findings, this research focuses on the management relationship between training effect and risk factors occurrence in engineering.

In the previous studies, the calculation of risk and effect assessment was often based on the experts' criteria and ignored the influence of subjective wills. For example, it is difficult to know whether an expert is sensitive to a certain influencing factor and therefore the objective rules of development are ignored in the scoring. Moreover, the common mathematical model evaluation methods do not take into account the randomness and ambiguity of the evaluated objects, so the evaluation results sometimes differ greatly from the real situation. With the reduction of China's demographic dividend, the policy of industrializing workers has become an excellent strategy for China to address the coming huge labor shortage. There is still a large research gap on how to ensure the quality of training and education for industrializing workers. To fill this gap and make a combination to the prospect of highly intelligent industrial development in the context of Industry 4.0, this paper combines AI machine vision with an evaluation system as a theoretical basis. The industrial worker training evaluation system is improved by considering the combined effect of workers' personal influence and experts' subjective will, so that the industrial worker training evaluation results can make timely and effective rectification of hidden engineering problems in the existing project management process of prefabricated buildings construction projects.

## 3. Methods

### 3.1. Face and LFER Facial Expression Recognition Based on Multitask Convolutional Neural Network

A large number of algorithms for image segmentation and artificial intelligence recognition already exist, and each algorithm is designed for different areas. For the background of this research, the MTCNN face recognition algorithm is selected for individual objects, and, because of the need for real-time evaluation of facial expressions, the Lighten Facial Expression Recognition (LFER) is chosen and improved as human facial expression recognition algorithm, and the FER2013 dataset is used for expression classification and recognition training.

#### 3.1.1. MTCNN Human Face Recognition Method

The high accuracy of the MTCNN face recognition algorithm comes from the organic combination of face detection and facial landmark detection. In the face classification and bounding-box regression stage, the main framework of MTCNN is similar to that of the Cascade algorithm and can be decomposed into a three-layer network structure. Before detection, the images are transformed to different sizes and image pyramids are constructed to accommodate face images of different pixels.

The MTCNN implementation process mainly adopts the method of candidate box plus classifier to realize fast and efficient face detection. The three cascade networks are P-Net for fast candidate box, R-Net for candidate box filtering selection, and O-Net for generating face landmarks and face boundaries [37].

The training data for the MTCNN algorithm was sourced from the Wider and CelebA databases. Wider gives the face detection data and marks the coordinate information of the bounding box, and CelebA provides the five landmark coordinate points [38].

*(1) P-Net*. The Proposal Network (P-Net) consists of a fully convolutional network. Rough feature extraction and boundary calibration of image pyramid are realized through fully convolutional networks (FCN), and the Nonmaximum Suppression (NMS) and bounding-box regression are used for border filtering and adjustment. The initial filtering transmits possible face region images determined by classifier to the next layer of the network.

In the screening process, each input sample should be represented by a cross-entropy loss function, which requires the use of positive and negative samples. The probability calculation formula for the face is shown in the following equation:

(1) $$\begin{array}{c} L_{i}^{\det}=-\left(y_{i}^{\det}\log\left(p_{i}\right)+\left(1-y_{i}^{\det}\right)\left(1-\log\left(p_{i}\right)\right)\right). \end{array}$$

*(2) R-Net*. The Refined Network consists of a convolutional neural network with an additional fully linked layer, which is more complex than the P-Net. The images transmitted from P-Net are further filtered and adjusted to achieve high-precision filtering and optimization of face image regions.

In the process of border regression, for each candidate box, the loss between the predicted offset and the true coordinate offset needs to be calculated. The formula for calculating the regression loss by Euclidean distance is shown in the following equation:

(2) $$\begin{array}{c} L_{i}^{\text{box}}={\left\|{\hat{y}}_{i}^{\text{box}}-y_{i}^{\text{box}}\right\|}_{2}^{2}. \end{array}$$

*(3) O-Net*. The Output Network has one more layer of convolutional neural network than R-Net and regresses the facial feature points of the filtered face image. The image features are saved in bulk to optimize the model performance. Finally, the coordinates of the top left and bottom right corners are output for plotting bounding boxes and five face image feature points are output.

Feature coordinate point location is similar to that of candidate box regression, in which the Euclidean distance between the candidate feature coordinate offsets and the true coordinate offsets needs to be calculated to obtain the overall loss function. The formula for calculating the regression loss from the Euclidean distance in the landmark sample and the overall loss function are shown in the following equation:

(3) $$\begin{array}{c} L_{i}^{\text{landmark}}={\hat{y}}_{i}^{\text{landmark}}-y_{i2}^{\text{landmark}2},\\ \min\sum_{j=1}^{N}\sum_{j\in \left\{\det,\text{box},\text{landmark}\right\}}\alpha_{j}\beta_{i}^{j}L_{i}^{j}. \end{array}$$

**Figure 1:**
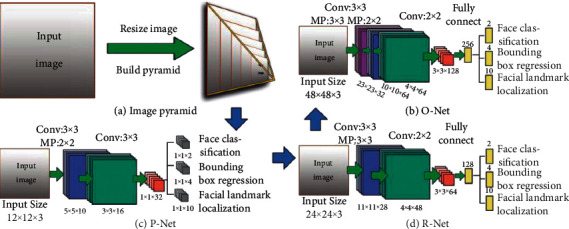
The image structure overlay and deconstruction of the P-Net, R-Net, and O-Net network structures. (a) Image pyramid. (b) O-Net. (c) P-Net. (d) R-Net.

An image structure overlay and deconstructed diagram of the three-layer network structure are shown in Figure 1.

#### 3.1.2. Lighten Facial Emotional Recognition Method

Human facial expression recognition relies on the classification of human face images collected by face recognition algorithms, equivalent to adding a classifier to the face recognition algorithm. LFER uses an annual deep convolutional network to fuse face expression feature extraction and classification into an end-to-end network. VGG-16/19 and ResNet-18/101/164 are employed in expression recognition and classification. Dropout is added before the full linked layer to increase the robustness of the algorithm model and prevent overfitting. An increase in robustness will improve the stability of the model and increase its recognition ability. Overfitting will lead to a lack of dispersion, thus increasing the probability of misjudgment. The seven categories of expressions were feeling disgusted, angry, sad, worried, neutral, happy, and surprised. The full linked layer in VGG and ResNet is removed, and the concept of loss function and cross-entropy is introduced. The calculation process of cross-entropy is shown in the following equation [39]:

(4) $$\begin{array}{c} J\left(\theta \right)=-\frac{1}{m}\sum_{i=1}^{m}\left[y^{i}\log\left(h_{\theta }\left(x^{i}\right)\right)+\left(1-y^{i}\right)\log\left(1-h_{\theta }\left(x^{i}\right)\right)\right]. \end{array}$$

The LFER algorithm uses Network Slimming and binarization methods for model compression to speed up recognition and employs Batch Normalization (BN) to reduce computation amount. The images of low BN indicator will be removed. The BN calculation formula is shown as follows:

(5) $$\begin{array}{c} \hat{z}=\frac{\left(z_{\text{in}}-\mu \beta \right)}{\sqrt{\sigma_{\beta }^{2}+\varepsilon };z_{\text{out}}=\gamma \hat{z}+\beta }. \end{array}$$

### 3.2. Structural Entropy Weight Method

In information theory, entropy represents the measure of uncertainty of a dataset. The absolute value of entropy becomes larger as the uncertainty of the information expressed in the dataset increases, and the value of entropy contains more information. Correspondingly, the smaller the value of entropy is, the poorer the information it contains and the more homogeneous the meaning of the representative indicator is.

Since SEWM has the function of both quantitative and qualitative analysis, it can be called an objective empowerment method in which the variation of values depends on the dispersion of the calculated dataset. The basic process of SEWM calculation is as follows: firstly, making a combination with Delphi method and fuzzy analysis method. The fuzzy analysis method is used to fuzzify the experts' scores and quantify the ranking according to the subjective will of the experts. After the quantification is completed, the normalization process is performed. Then, cognitive blindness degree analysis and entropy calculation are performed for the quantitative order of experts' judgments, and the possible bias due to the subjective opinions of experts is corrected. Finally, the weighting value is calculated [40]. The detailed calculation and formula are shown below.

#### 3.2.1. Arranging the Quantization Order of the Datasets

According to the Delphi method, *k* experts in the relevant fields are selected to fill out the questionnaire. The basis of data acquisition in this paper is built on machine vision, so the sample data of answers from *k* relevant workers can be used as the data source. The questionnaire taken by the original Delphi method is replaced by a random fixed number of classified questions from the question bank.

The importance of the sample data is obtained by the positive and negative selection method considering facial expressions in this paper, where 1 is an important indicator and *n* unimportant. For the *i* samples and *j* indicators in the dataset, where 1 ≤ *i* ≤ *k* and 1 ≤ *j* ≤ *n*, the representation performed by the  *a*_*ij*_ array is shown as follows:

(6) $$\begin{array}{c} a_{\text{ij}}=\left[\begin{array}{ccc} a_{11} & \cdots & a_{1n}\\ \vdots & \ddots & \vdots \\ a_{k1} & \cdots & a_{kn} \end{array}\right]. \end{array}$$

#### 3.2.2. Making Normalization of Indicators: Homogenization of Heterogeneous Indicators

Each indicator may have inconsistent measurement units in statistical decision-making, so it is necessary to standardize the sorted datasets and convert the relative values of indicators into absolute values to solve inconsistent measurement methods for different indicators. Since the normalization of the data group value is an absolute value, the greater the difference in the Hamming distance of each normalized data, the greater the dispersion. The normalization processing method is related to the calculation method, as shown in (7) and (8):

Positive indicators:
  (7) $$\begin{array}{c} a_{\text{ij}}^{'}=\frac{a_{\text{ij}}-\min\left\{a_{1j},a_{2j},\ldots ,a_{\text{nj}}\right\}}{\max\left\{a_{1j},a_{2j},\ldots ,a_{\text{nj}}\right\}-\min\left\{a_{1j},a_{2j},\ldots ,a_{\text{nj}}\right\}}. \end{array}$$
Negative indicators:
  (8) $$\begin{array}{c} a_{\text{ij}}^{'}=\frac{\max\left\{a_{1j},a_{2j},\ldots ,a_{\text{nj}}\right\}-a_{\text{ij}}}{\max\left\{a_{1j},a_{2j},\ldots ,a_{\text{nj}}\right\}-\min\left\{a_{1j},a_{2j},\ldots ,a_{\text{nj}}\right\}}. \end{array}$$

To simplify the formula, the normalized data *a*_ij_′ is still denoted by *a*_ij_.

#### 3.2.3. Weight Calculation for Qualitative Analysis and Quantitative Analysis

Relying on the concept of entropy weight method, the quantitative analysis data is revised, where *I* is the order in the data group and *m* is the conversion parameter whose values are usually equal to *j*+2 (*m*=*j*+2) [40]. The quantitative analysis correction is shown as follows:

(9) $$\begin{array}{c} \mu \left(I\right)=-\frac{\ln\left(m-I\right)}{\ln\left(m-1\right)}. \end{array}$$

Substituting *a*_ij_ into the above formula, the quantitative analysis correction array *b*_ij_ is obtained. The expert cognitive blindness degree analysis parameter *Q*_*j*_ is introduced. The calculation formula of *Q*_*j*_ is shown as follows:

(10) $$\begin{array}{c} Q_{j}=\left|\frac{\left\{\left[\max\left\{b_{1j},b_{2j},\ldots ,b_{\text{nj}}\right\}-b_{j}\right]+\left[\min\left\{b_{1j},b_{2j},\ldots ,b_{\text{nj}}\right\}-b_{j}\right]\right\}}{2}\right|. \end{array}$$

The perception of the relevant respondents for the indicator is defined as *x*_*j*_ and the standardized weights are calculated. The calculation process is shown in the two following equations:

(11) $$\begin{array}{c} x_{j}=b_{j}\left(1-Q_{j}\right). \end{array}$$

(12) $$\begin{array}{c} \omega_{j}=\frac{x_{j}}{\sum_{j=1}^{n}x_{j}}. \end{array}$$

### 3.3. Similarity Aggregation Method

For the possibility of fuzzy numbers and uniform or normal distribution, the linguistic terms and sentiment labels of the relevant descriptions are expressible as affiliation functions of different values. In previous risk assessments, triangular and trapezoidal fuzzy numbers were mostly used. For qualitative and comparative analysis, converting collected data into fuzzy numbers is more adaptable than converting them into probability distributions [41]. In this study, it is necessary to use the improved SAM to convert the data into fuzzy numbers for aggregation and then to evaluate the effect through weight calculation.

This study proposes an improved SAM that introduces the concept of emotional expression when inputting data and changes the previous expert evaluation system to the facial expression recognition dataset of the surveyed person. The method quantifies the change in respondents' emotions during the test through human behaviour modelling, and the resulting fuzzy numbers are corrected based on multiple factors. In previous studies, some scholars selected experts' position, duration of practice, age, and education as rank divisions [42].

The improved SAM calculation processes are as follows:

(1) Suppose that there are *E*_*i*_ experts, where 1 ≤ *i* ≤ *k*. Calculate the consistency $S\left(\tilde{R_{u}},\tilde{R_{v}}\right)$ of quantitative data for each expert (respondent), where *R*_*u*_ and *R*_*v*_ are the fuzzy numbers of *S*. The formula of quantitative data consistency is shown as follows:
  (13) $$\begin{array}{c} S\left(\tilde{R_{u}},\tilde{R_{v}}\right)=1-\frac{1}{5\sum_{i=1}^{5}\left|a_{i}-b_{i}\right|}, \end{array}$$
*a*_*i*_ and *b*_*i*_ represent the evaluation parameter values of different qualitative analysis. The larger the *S* value, the more unified the opinions of experts (respondents).
(2) Subsequently, the Delphi method was used for secondary quantitative analysis, combined with credibility to improve the reliability of aggregated analysis results. Finally, the consistency WA(*E*_*u*_) of weighted agreement (absolute value) is calculated, and the relative consistency RA(*E*_*u*_) and the consistency coefficient RC(*E*_*u*_) of the expert dataset are calculated according to the expert's WA(*E*_*u*_). In previous studies, some scholars introduced the concept of relaxation factor *δ*, *δϵ*[0,1]. When the value of *δ* approaches 1, it means that the experts' judgment weight is the main basis; when the value of A tends to 0, it means that RC(*E*_*u*_) is an indicator of the relative value of experts' judgments. Finally, the summary results of experts' opinions are calculated to obtain the overall fuzzy number $\tilde{R}$. The calculation formula of $\tilde{R}$ is
  (14) $$\begin{array}{c} \tilde{R}=\sum_{u=1}^{M}\left[\text{RC}\left(E_{u}\right)\times \tilde{R_{u}}\right]. \end{array}$$

## 4. Data Survey and Model Establishment

### 4.1. Data Investigation and Collection

The data for this study, done with the assistance of Python 3.8 programming language, were obtained from the results of the responses to the questions drawn from the question bank, which were all multiple-choice questions about knowledge related to safety in assembled buildings, divided into five sections: Knowledge about Engineering Project Management (KPM), Cautions at Construction Site (CCS), Prefabricated Component Hoisting and Installation (PHI) Knowledge, Emergency Accident Handling Methods (EAM), and Laws and Regulations Related to Construction (LRC). As respondents took the training test, cameras were used to capture their facial expressions, recorded at 30 frames per second, with a 10-second response time for each question and a final 2-second answer checking phase. The footage is then split at 15-frame intervals. Twenty face images are collected for each question, and each respondent can provide 2000 face images for analysis. The pseudocode of collecting facial expression data for the industrializing worker training test is shown in Algorithm 1.

**Figure 2:**
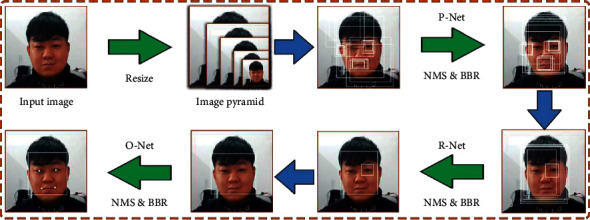
Face image recognition and feature calibration process.

**Figure 3:**
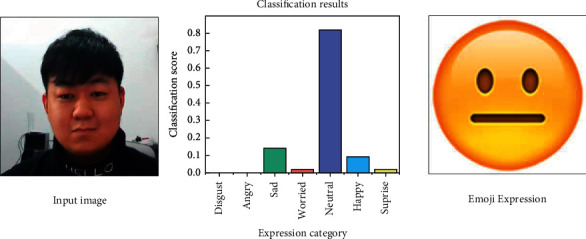
The result of facial expression judgment.

Subsequently, every 20 images were formed into a data group for expression analysis using the MTCNN-LFER model. Firstly, the acquired images are decomposed into image pyramids and then imported into the three-layer volume and network P/R/O-Net for expression analysis and recognition. The recognition process is shown in Figure 2. Then the probability of emotions is determined based on the relative coordinates of the expression features, and the output of the determination is shown in Figure 3.

### 4.2. Evaluation Model of Facial Expression Recognition for Industrializing Worker Training

**Figure 4:**
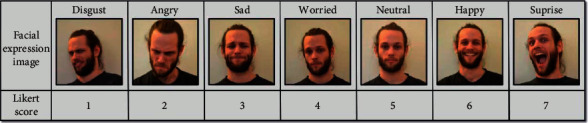
Expression score division based on Likert's seven-level scale.

Quantitative analysis of the collected data is required before the construction of the expression recognition evaluation model. The difference between the data collection phase of this study and the establishment of previous evaluation models is that previous evaluation models mostly use the mechanism of expert scoring, while that in this study is derived from the respondents' status while taking the answer test. This study uses the Likert's seven-level scale method to assign different categories of facial expression recognition (including disgusted, angry, sad, worried, neutral, happy, and surprised) from 1 to 7 points. The formula for preliminary scoring is shown in Equation (15). *t* is the moment, *p*_emo_ is the bias probability for each expression, and *S*_*l*7_ is the assigned score on the 7-point Likert scale. *S*_*r*_ is the parameter for correct or incorrect answers, and when the answer is correct, *S*_*r*_ is 1.2; otherwise, *S*_*r*_ is 0.8. The division of expression scores based on Likert's seven-level scale is shown in Figure 4 [43].

(15) $$\begin{array}{c} W_{\text{emo}}=\sum_{t=1}^{20}\sum_{p_{\text{emo}}=1}^{7}\left(\frac{p_{\text{emo}}\times S_{l7}}{n}\right)S_{r}. \end{array}$$

However, the assignment of expressions is not the final “expert score,” because the data comes from training tests, and the answers will be right or wrong. However, in the process of testing, workers may be unskilled in the operation of electronic equipment or emotionally resulting in deviations in the visual area. Furthermore, there is a possibility that when a worker encounters an uncertain option, he guesses the correct option by luck. Monaro et al. [24] defined the concept of error and hesitation degree in the research of facial expression detection deception. Through the survey, it was found that, due to differences in education, gender, age, and other influencing factors, different people will have abnormal facial expressions when they make judgment. According to the average conditions of Chinese workers, the range of the error degree *τ* is 0.6514 ± 0.1343, and that of hesitation degree *υ* is 0.4875 ± 0.098 [24].

**Figure 5:**
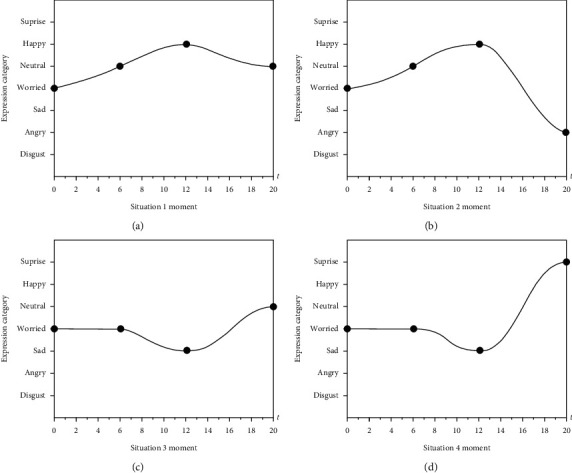
Diagram of four abnormal mood fluctuations. (a) Situation 1. (b) Situation 2. (c) Situation 3. (d) Situation 4.

In view of the above situation, this article expresses the worker's facial expression changes and corresponding moments of each question with images and selects 4 curved surfaces and curves under abnormal mood fluctuations as special images for analysis. The four abnormal images are shown in Figure 5. According to the fluctuation trend of the four curves, it can be analyzed as the four following situations.

**Situation 1 .**

When the respondents first saw the topic, the expression was worried, indicating that the worker did not understand the randomly selected topic and felt worried. But, in the subsequent reading stage, workers who are skilled in training knowledge tend to be natural in their expressions. After finishing reading the questions, if the workers have a good memory of the correct answers, their facial expressions will turn into a happy state and will turn into a natural look during the answer verification stage.

**Situation 2 .**

Before the answer verification, it is the same as situation 1. When checking the answers, if the worker is fully sure of the knowledge points involved in the question, but when making choices, it may be due to operational errors or incorrect memory, which may cause the worker to have a short period of frustration during the test. In order to eliminate the worker's wrong operation and memory error, the error degree *τ* (*τ*=0.7) is introduced for the first time to correct the evaluation score of the worker's answers.

**Situation 3 .**

When workers are not very familiar with randomly selected questions, they will maintain a worried look at the initial reading stage; then when thinking about the questions further, they will show sadness and regret expressions due to the vague memory. In the answer verification stage, as the wrong answer is expected, the worker's expression will return to a sad look, worrying about the next question.

**Situation 4 .**

Before the answer verification stage, it is similar to situation 3. When checking the answers, they will feel surprised because of the unexpected correct answers. However, the workers' choices under this emotion are only done through luck and guessing, so this study introduces hesitation degree *υ* (*υ*=0.45) to modify the evaluation score of the workers' answers [44].

**Figure 6:**
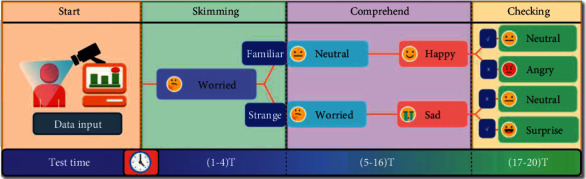
Evaluation process of sentiment analysis of industrializing worker training test.

Based on the four situations of emotional changes in industrializing workers training test under normal conditions, this paper establishes an emotional analysis and evaluation process for industrializing workers training test. The process is shown in Figure 6.

According to process analysis, (15) is improved with the introduction of the concepts of error and hesitancy degree. The improved expression score division weight calculation method is shown as follows:

(16) $$\begin{array}{c} W_{\text{emo}}=\left(\overset{4}{\underset{t=1}{\sum }}\overset{7}{\underset{p_{\text{emo}}=1}{\sum }}\left(\frac{p_{\text{emo}}\times S_{l7}}{n}\right)+\overset{16}{\underset{t=5}{\sum }}\overset{7}{\underset{p_{\text{emo}}=1}{\sum }}\left(\frac{p_{\text{emo}}\times S_{l7}}{n}\tau \right)+\overset{20}{\underset{t=17}{\sum }}\overset{7}{\underset{p_{\text{emo}}=1}{\sum }}\left(\frac{p_{\text{emo}}\times S_{l7}}{n}\upsilon \right)\right)S_{r}. \end{array}$$

All data collected in this study were entered into the evaluation process, and the results were calculated with SEWM-SAM to derive the final weight ranking. The highest weight value is the most significant point of the model source data.

## 5. Result

This survey study relies on a prefabricated building project at Langfang Airport Free Trade Port, which is implementing a career path promotion for industrializing workers. After training and reeducation of the workers, a worker who had recently violated engineering regulations was selected and surveyed and analyzed by using the model developed in this paper. The testing system in this study draws 100 questions at a time from a pool of safety knowledge questions, with 20 questions in each of the five professional directions. Two thousand images and 14,000 cells of analysis data were collected for a single worker.

### 5.1. Facial Expression Recognition Score Based on MTCNN-LFER

**Figure 7:**
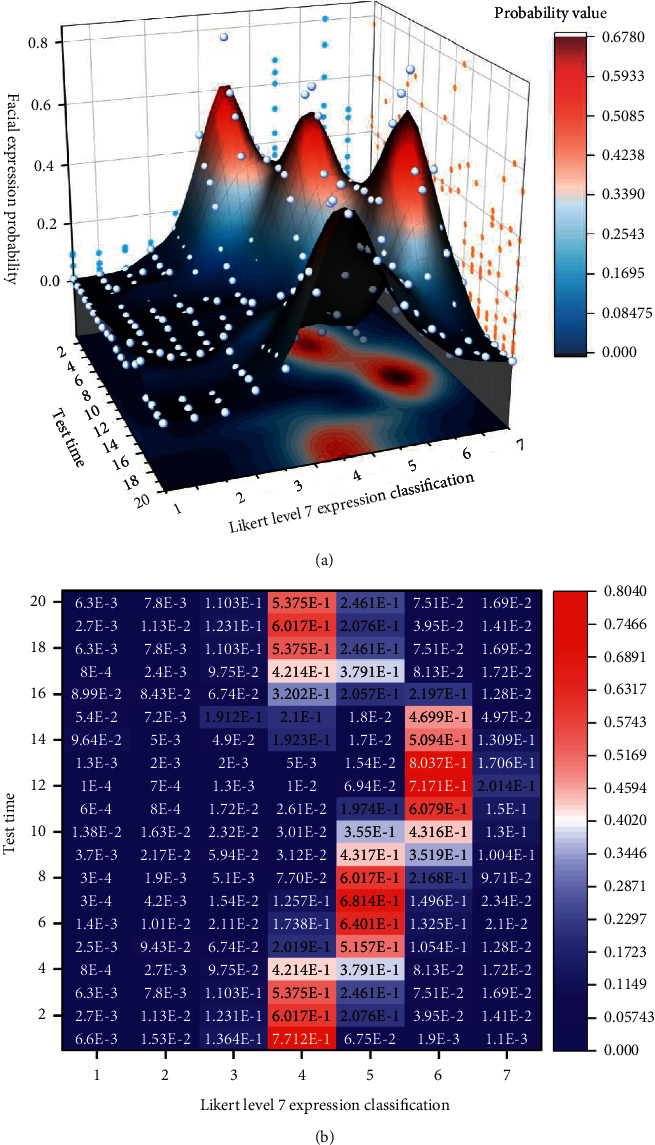
Facial expression analysis and data extraction process. (a) Surface map. (b) Determining the numerical thermal distribution map.

Taking the multiple-choice questions in the construction site precautions test as an example, the collected images of respondents' expressions at that time were analyzed and imported into the facial expression recognition evaluation model for industrializing worker training in this study for score evaluation. According to the grading level of the education industry, 75% of the highest value is taken as the pass line. The process of image analysis and data extraction imported into the model is shown in Figure 7. The facial expression analysis data are shown in Table 1.

The data scores of 20 moments for each question were brought into the model of this paper and calculated by equation (16) to get *W*_emo_ value for each question. The values are shown in Table 2.

Find the average of *W*_emon_, which is $\bar{W_{\text{emon}}}$ of the current question, indicating the average value of the standard scoring of this question in the evaluation system, which can be used as SWEM expert scoring data.

### 5.2. Numerical Solution of SEWM Weight

The test questions were graded into 5 first-level indicators and 20 second-level indicators by analyzing the collected image expression emotion data. The 5 first-level indicators are KPM, CCS, PHI, EAM, and LRC, and the 20 second-level indicators correspond to the questions under each first-level indicator. The expression scores of different moments represent the scoring values of experts. The weights of second-level indicators related to Section A are shown in Table 3.

After calculating the weights of the second-level indicators of the five sections, the values are used as the reference data for conducting the comparative solution. First, the extreme values of the second-level indicators' weights are transformed into fuzzy arrays, whose classifications are shown in Table 4. The weight of a single second-level indicator is multiplied by the corresponding grade score to find the average value, and the approximate integer solutions are the data of the first-level indicators. Then the data are substituted into equations (6)–(12) to get the weights of the first-level indicators. The weights of the first-level indicators are shown in Table 5.

From the first level and second level of indicator weight values, it can be analyzed that the expression data plays a decisive role in the evaluation of each question, and the change of the values can be divided into 3 segments, where the middle segment has the greatest influence, and when the good emotion overlaps with the correct option, the final comprehensive weight value will be larger.

### 5.3. Analysis of the Significance of SAM

#### 5.3.1. Establishing Linguistic Fuzzy Sets

In this study, five levels of fuzzy classification languages are used for expert inspiration. The fuzzy linguistic number set is determined by trapezoidal fuzzy numbers. The hazard impact assessment levels are shown in Table 6. In this study, 20 questions in each section are used to assess the understanding of surveyed workers on the 5 sections, and the total fuzzy number is derived from the formula to measure the implementation effect of industrializing worker training, where the relative weights of the sections are equal to the weights of the first-level indicators of each section.

The fuzzy language set of the expert evaluation classification is substituted into (13) as the parameters *a*_*i*_ and *b*_*i*_, and the evaluation data of each section are aggregated through SAM. Because the relaxation factor requires a large number of actual events to be tested, as a new method proposed for the training of industrializing workers, this study refers to relevant literature and takes the general value of the relaxation factor *β*=0.5 as the test.

#### 5.3.2. Acquiring Aggregation Fuzzy Numbers

A calculation of the consistency of the fuzzy language scale array to which each section belongs is made. The calculation process is as follows: $S\left(\tilde{R_{1}},\tilde{R_{2}}\right)=1-\left[\left(0.4-0.2\right)+\left(0.4-0.3\right)+\left(0.5-0.4\right)+\left(0.6-0.4\right)+\left(0.6-0.5\right)\right]/5=0.86$. The results are substituted into the calculation of the weighted agreement degree WA(*E*_*n*_) and the relative agreement degree RA(*E*_*n*_). Then the relaxation factor is used to calculate the expert agreement coefficient, for example, CC(*E*_1_)=0.5 × 0.3706+(1 − 0.5) × RA(*E*_1_). Substituting the expert weighted agreement degree, relative agreement degree, and agreement coefficient into (14), the overall fuzzy number *R* can be calculated. The calculation data of experts' opinions fuzzy aggregation indicators are shown in Table 7.

**Figure 8:**
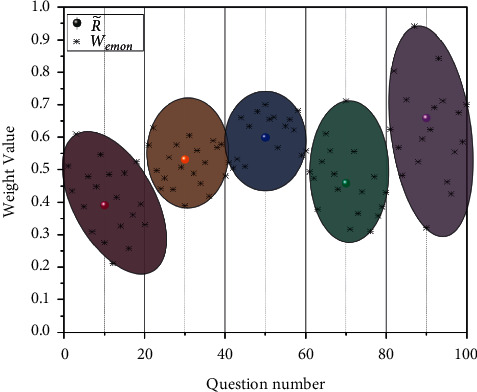
Cluster analysis of industrializing worker training management system.

Through aggregation calculation, the aggregation total fuzzy number $\tilde{R}=\left(0.3909,0.5309,0.5990,0.4590,0.6590\right)$ of expert opinion aggregation is obtained. The aggregated total fuzzy number and the second-level indicators are made into a cluster analysis, as shown in Figure 8. According to the images of the data group, it can be seen from the feedback from the training test of industrializing workers that the worker has the worst knowledge of relevant construction laws and regulations, which is in line with the fact that he has made mistakes in the project funds recently.

According to the statistics of the correct rate of the questions in the 5 sections, the correct rates of the questions in Section 2, 3, and 5 are all more than 75%, indicating that the worker has passed the test in these areas. However, with the score gap in Section 5 being too huge, it is necessary to verify whether it is personal reasons or a faulty training system that caused this result.

### 5.4. Model Validation

**Figure 9:**
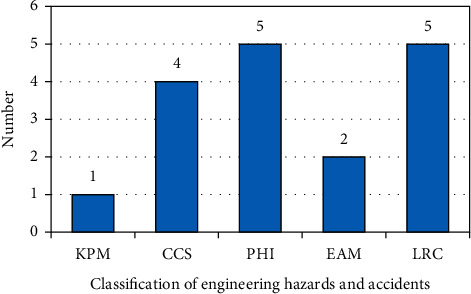
Statistical graph of the number of hidden dangers and accidents in the Langfang Airport Free Trade Port prefabricated building projects.

This study relies on a prefabricated building project at Langfang Airport Free Trade Port and conducts surveys and statistics after the construction of the project. A total of 17 engineering hidden dangers and accidents were found in the acceptance stage. The 17 hidden dangers and accidents were classified according to the 5 first-level indicators in this study. The classified statistical graph of the number of hidden dangers and accidents in the Langfang Airport Free Trade Port prefabricated building projects is shown in Figure 9.

In this study, 10 workers who participated in the construction of prefabricated buildings in Langfang Airport Free Trade Port were randomly selected for training effect assessment, and the results were calculated. The aggregated total fuzzy number values are shown in Table 8.

**Figure 10:**
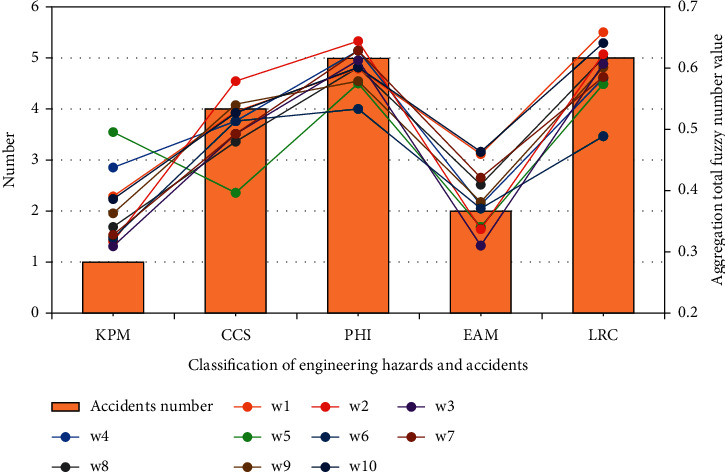
Association chart between industrializing workers' training assessment results and potential dangers and accidents in Langfang Free Trade Port project.

The aggregated total fuzzy number of training assessment of 10 workers was compared with the number of engineering hidden dangers and accidents in the acceptance stage of Langfang Airport Free Trade Port. The association chart between industrializing workers' training assessment results and potential dangers and accidents in Langfang Free Trade Port project is shown in Figure 10.

From the comparison in the figure, it can be found that the aggregated total fuzzy number value of the worker's training assessment results is positively correlated with the probability of occurrence of such hidden dangers and accidents in the engineering projects that the worker is engaged in. As can be seen from Figure 10, the group of workers in the project at Langfang City Airport Free Trade Port was not well trained in CCS, PHI, and LRC, so the project has many hidden dangers and accidents in the acceptance stage, with 4, 5, and 5 cases, respectively, which proves the correctness of the model proposed in this study.

## 6. Discussion and Conclusion

### 6.1. Discussion

In the process of building the model, MTCNN-LFER was used to analyze the respondents' facial expressions in real time in order to conform to objective facts, and four situations were analyzed according to the characteristics of expression changes under different mental activities. In previous evaluation system, respondents' feedback was only scored based on their performance results. For example, in Scenario 2, the respondents' misselection would be directly judged as a wrong situation according to the conventional evaluation system. However, their initial judgments are valid. Inaccurate evaluation results will lead to wrong final rating, thus affecting workers' training enthusiasm. For construction units, wrong evaluation leads to redundant training course arrangement, which affects the efficiency of workers' industrialization process and also wastes a lot of financial and material resources [33].

However, there is another situation, such as Situation 4, where the worker guesses the correct option. For this case, the traditional evaluation method does not consider whether the worker has a solid knowledge of the question but only recognizes that the worker's choices are correct and thus determines that the worker has a high level of mastery of such knowledge. According to the description of human behaviour, humans always make constant rather than consistent and repetitive guesses about the unknown. The worker will choose another answer when the correctly guessed question is retested in a short time. For workers, with the help of good luck to save time and effort, they do not need to master engineering-related knowledge. However, for construction units, workers who muddle through with this trick may cause greater property damage or even affect engineering safety and cause malignant casualty accidents. Therefore, it is necessary to introduce relevant parameters to modify the model to ensure that the model is suitable for the development rule of objective facts, so that the applicability of the evaluation system is stronger, which will have guiding significance for similar industries.

According to the final weight calculation and cluster analysis results, it was found that, in some knowledge sections, although a small number of them scored high and the final composite score was higher compared with other sections, there was a large dispersion, which proved that workers did not have a firm grasp of such knowledge and still many knowledge points needed to be checked and remedied. Therefore, even though the overall score is the highest, there is still a need for training and education. In engineering construction, muddling through is never an option, as workers with poor knowledge may have a great impact on the entire project. In Li et al.'s study, it was noted that unsafe behaviour in engineering is related not only to the workers' own behaviour but also to cognitive biases in different contexts. Poor worker handling can also contribute to engineering accidents; however, the correctness of the handling is related to the way the worker chooses to deal with the problem [45].

The weight solution of this study for each question can also give feedback on the proficiency of the question in the section to which it belongs; in particular, the test after training can reflect whether workers have evasive behaviour for a certain knowledge point. Chen has suggested in previous research that workers will often adopt unsafe behaviours to ensure that progress is efficient [46]. This type of problem can be explained by the pattern presented by the group industrial worker training test to explain the current engineering safety climate problem that exists in the group of industrial workers.

For example, a worker has insufficient knowledge about construction phase reports when answering questions, and most of the workers in the construction team have this situation; then it can be inferred that the construction is in poor quality. The root of this can be found to be a hidden line of conflict of interest between the worker, the construction unit, and the regulator [47]. The quality of training of the group can thus be screened to identify more hidden dangers that affect the economy and safety of engineering under the policy of industrializing worker training process.

According to the model validation, it can be seen from the calculation and analysis of the evaluation results of the training management of industrializing workers in this study that hidden dangers and accidents in engineering projects are closely related to the skills mastered by workers. In all construction stages of engineering projects, the management of industrializing worker training can be improved to eliminate possible hazards and stop accidents in time to ensure safe, economical, and stable construction and production of the engineering project.

### 6.2. Conclusion

This paper is based on the background that the Chinese government proposes to accelerate the process of industrializing workers in response to the rapid reduction of the domestic demographic dividend and the rapid weakening of the labor advantage in the era of Industry 4.0. The artificial intelligence machine vision and fuzzy mathematical evaluation system are combined to test the workers who receive industrializing training and record the information related to the change of facial expressions and the correct rate of assessment when the workers receive the test assessment. In addition, the evaluation model of facial expression recognition for industrializing worker training is used to conduct qualitative and quantitative analysis and finally arrive at the aggregated information results. The main conclusions of this study are as follows:

1. The expert scoring mechanism in the evaluation model is improved by using machine vision to analyze the changes in workers' facial expressions during the test to determine their mental activities, effectively preventing the waste of money and time for subsequent training due to wrong operations. It also prevents workers from guessing the answers and ignoring the forgotten knowledge points, which reduces the possibility of more safety accidents and property damage to the project.
2. The questions of each section are converted into primary and secondary indicators to conduct an aggregation consistency analysis, and the weight of each indicator and the significance of the aggregation analysis are used to indicate the degree of mastery of the tested workers on the knowledge of each section. According to the discrete degree of each question, we can check whether it is a common problem of the construction workers and can timely find out the safety, economic, or management hazards.

In this paper, although the necessity of objective facts is considered to the greatest extent when analyzing and mathematically modelling the changes in facial expressions of workers doing the test, there still exist the following deficiencies:

1. There is no evaluation or research analysis of workers' psychological states. Actually, the differences in personalities and psychological states may affect the accuracy of emotions conveyed through facial expressions during the test.
2. In analyzing the dispersion of each knowledge point, only the problems of the workers were considered, but, in fact, it is possible that some irregularities caused by “traditional experience” of the industry lead to workers not recognizing the standardized operation, or some people with management authority forbid workers to follow the standardized practice of construction or project handling.

These deficiencies can be refined in future research and solved by multiobjective optimization methods.

## Figures and Tables

**Algorithm 1:**
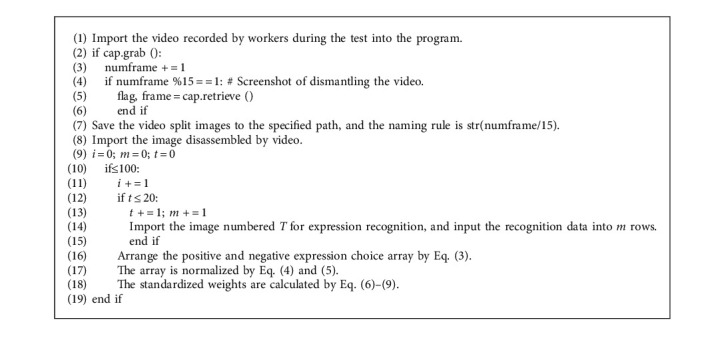
Pseudocode of facial expression data collection for industrializing worker training test.

**Table 1:** Facial expression analysis data.

Time	Disgusted	Angry	Sad	Worried	Neutral	Happy	Surprised
1	0.0066	0.0153	0.1364	0.7712	0.0675	0.0019	0.0011
2	0.0027	0.0113	0.1231	0.6017	0.2076	0.0395	0.0141
3	0.0063	0.0078	0.1103	0.5375	0.2461	0.0751	0.0169
4	0.0008	0.0027	0.0975	0.4214	0.3791	0.0813	0.0172
5	0.0025	0.0943	0.0674	0.2019	0.5157	0.1054	0.0128
6	0.0014	0.0101	0.0211	0.1738	0.6401	0.1325	0.0210
7	0.0003	0.0042	0.0154	0.1257	0.6814	0.1496	0.0234
8	0.0003	0.0019	0.0051	0.0771	0.6017	0.2168	0.0971
9	0.0037	0.0217	0.0594	0.0312	0.4317	0.3519	0.1004
10	0.0138	0.0163	0.0232	0.0301	0.3550	0.4316	0.1300
11	0.0006	0.0008	0.0172	0.0261	0.1974	0.6079	0.1500
12	0.0000	0.0007	0.0013	0.0101	0.0694	0.7171	0.2014
13	0.0012	0.0020	0.0020	0.0051	0.0154	0.8037	0.1706
14	0.0964	0.0050	0.0490	0.1923	0.0170	0.5094	0.1309
15	0.0539	0.0072	0.1912	0.2101	0.0180	0.4699	0.0497
16	0.0899	0.0843	0.0674	0.3202	0.2057	0.2197	0.0128
17	0.0008	0.0027	0.0975	0.4214	0.3791	0.0813	0.0172
18	0.0063	0.0078	0.1103	0.5375	0.2461	0.0751	0.0169
19	0.0027	0.0113	0.1231	0.6017	0.2076	0.0395	0.0141
20	0.0063	0.0078	0.1103	0.5375	0.2461	0.0751	0.0169

**Table 2:** *W*_emon_ value considering the error degree, hesitation degree, and correctness rate.

Time (*t*)	*W*_emon_	Time (*t*)	*W*_emon_	Time (*t*)	*W*_emon_	Time (*t*)	*W*_emon_
1	0.5554	6	0.4922	11	0.5842	16	0.4177
2	0.5964	7	0.5026	12	0.61049	17	0.2885
3	0.6146	8	0.5316	13	0.6125	18	0.2765
4	0.6411	9	0.5322	14	0.5080	19	0.2684
5	0.4501	10	0.5511	15	0.4739	20	0.2765

*Note*.*τ*=0.7;  *υ*=0.45;  *S*_*r*_=1.2.

**Table 3:** The weights of second-level indicators related to Section A.

No.	Disgusted	Angry	Sad	Worried	Neutral	Happy	Surprised
1	0.0066	0.0153	0.1364	0.7712	0.0675	0.0019	0.0011
2	0.0027	0.0113	0.1231	0.6017	0.2076	0.0395	0.0141
3	0.0063	0.0078	0.1103	0.5375	0.2461	0.0751	0.0169
4	0.0008	0.0027	0.0975	0.4214	0.3791	0.0813	0.0172
5	0.0025	0.0943	0.0674	0.2019	0.5157	0.1054	0.0128
6	0.0014	0.0101	0.0211	0.1738	0.6401	0.1325	0.0210
7	0.0003	0.0042	0.0154	0.1257	0.6814	0.1496	0.0234
8	0.0003	0.0019	0.0051	0.0771	0.6017	0.2168	0.0971
9	0.0037	0.0217	0.0594	0.0312	0.4317	0.3519	0.1004
10	0.0138	0.0163	0.0232	0.0301	0.3550	0.4316	0.1300
11	0.0006	0.0008	0.0172	0.0261	0.1974	0.6079	0.1500
12	0.0000	0.0007	0.0013	0.0101	0.0694	0.7171	0.2014
13	0.0012	0.0020	0.0020	0.0051	0.0154	0.8037	0.1706
14	0.0964	0.0050	0.0490	0.1923	0.0170	0.5094	0.1309
15	0.0539	0.0072	0.1912	0.2101	0.0180	0.4699	0.0497
16	0.0899	0.0843	0.0674	0.3202	0.2057	0.2197	0.0128
17	0.0008	0.0027	0.0975	0.4214	0.3791	0.0813	0.0172
18	0.0063	0.0078	0.1103	0.5375	0.2461	0.0751	0.0169
19	0.0027	0.0113	0.1231	0.6017	0.2076	0.0395	0.0141
20	0.0063	0.0078	0.1103	0.5375	0.2461	0.0751	0.0169
*b*_11_	2.1117	2.1116	2.1056	2.0812	2.0819	2.0848	2.1067
*Q*_11_	1.0576	1.0575	1.0542	1.0463	1.0446	1.0507	1.0556
*x*_11_	0.1217	0.1214	0.1140	0.0963	0.0929	0.1057	0.1171
*w*_11_	0.1582	0.1579	0.1483	0.1252	0.1208	0.1374	0.1522

**Table 4:** The classification of the fuzzy array of the secondary indicator mapping.

Numerical interval	Level classification score
(0.1200,0.1300]	5
(0.1300,0.1400]	4
(0.1400,0.1500]	3
(0.1500,0.1600]	2
(0.1600,0.1700]	1

**Table 5:** The weight values of the first-level indicators.

Name	KPM	CCS	PHI	EAM	LRC	Name	KPM	CCS	PHI	EAM	LRC
Data1	3	4	1	2	2	Data11	5	3	3	4	2
Data2	4	3	2	1	3	Data12	4	5	5	3	1
Data3	4	2	1	3	1	Data13	3	4	3	3	3
Data4	4	2	1	3	3	Data14	4	2	2	5	4
Data5	4	4	2	3	2	Data15	1	5	5	5	2
Data6	3	2	1	4	4	Data16	5	5	4	4	3
Data7	4	5	1	3	5	Data17	5	4	2	3	4
Data8	3	3	2	1	2	Data18	3	3	4	4	2
Data9	2	4	1	3	1	Data19	4	4	3	5	1
Data10	1	4	1	4	3	Data20	2	1	2	3	2
*b*_*t*_	1.5266	1.5063	1.7968	1.5375	1.7030	*w*_1*t*_	0.3803	0.2078	0.0584	0.3681	0.0145
*Q*_*t*_	0.7200	0.8449	0.9635	0.7309	1.0096	Rank	1	3	4	2	5
*x*_*t*_	0.4274	0.2336	0.0656	0.4137	0.0163						

**Table 6:** Scale table of fuzzy language set.

Fuzzy numbers	Linguistic terms	Corresponding sections
Slight influence	（0, 0, 0.1, 0.2, 0.2）	—
Mild influence	（0.2, 0.3, 0.4, 0.4, 0.5）	1, 5
Moderate influence	（0.4, 0.4, 0.5, 0.6, 0.6）	2
Severe influence	（0.5, 0.6, 0.6, 0.7, 0.8）	4
Significant influence	（0.8, 0.8, 0.9, 1, 1）	3

Note: “—” means none.

**Table 7:** Fuzzy aggregation indicators calculation data of experts' opinions.

Variable name	Value	Variable name	Value
WA(*E*_1_)	0.7465	RA(*E*_4_)	0.2022
WA(*E*_2_)	0.8414	RA(*E*_5_)	0.2213
WA(*E*_3_)	0.5966	CC(*E*_1_)	0.2838
WA(*E*_4_)	0.7660	CC(*E*_2_)	0.2123
WA(*E*_5_)	0.8387	CC(*E*_3_)	0.1072
RA(*E*_1_)	0.1970	CC(*E*_4_)	0.2804
RA(*E*_2_)	0.2220	CC(*E*_5_)	0.1163
RA(*E*_3_)	0.1574		

**Table 8:** Aggregate total fuzzy number values for the training assessment of the 10 workers.

	KPM	CCS	PHI	EAM	LRC
$\tilde{R_{1}}$	0.3909	0.5309	0.5990	0.4590	0.6590
$\tilde{R_{2}}$	0.3167	0.5792	0.6451	0.3367	0.6237
$\tilde{R_{3}}$	0.3094	0.4920	0.6142	0.3105	0.6064
$\tilde{R_{4}}$	0.4380	0.5134	0.6279	0.3751	0.5834
$\tilde{R_{5}}$	0.4957	0.3978	0.5764	0.3417	0.5739
$\tilde{R_{6}}$	0.3227	0.5142	0.5342	0.3697	0.4896
$\tilde{R_{7}}$	0.3275	0.4935	0.6302	0.4217	0.5860
$\tilde{R_{8}}$	0.3410	0.4792	0.6017	0.4100	0.6176
$\tilde{R_{9}}$	0.3639	0.5406	0.5798	0.3817	0.6010
$\tilde{R_{10}}$	0.3867	0.5273	0.6014	0.4630	0.6427

## Data Availability

The case analysis data used to support the findings of this study are available from the corresponding author upon request.
